# The 30-days hospital readmission risk in diabetic patients: predictive modeling with machine learning classifiers

**DOI:** 10.1186/s12911-021-01423-y

**Published:** 2021-07-30

**Authors:** Yujuan Shang, Kui Jiang, Lei Wang, Zheqing Zhang, Siwei Zhou, Yun Liu, Jiancheng Dong, Huiqun Wu

**Affiliations:** 1 Department of Medical Informatics, Medical School of Nantong University, 19 Qixiu Road, Nantong, 226001 Jiangsu People’s Republic of China; 2 Department of Statistics and Data Management, Children’s Hospital of Fudan University, Shanghai, 201102 People’s Republic of China; 3 Department of Information, the First Affiliated Hospital, Nanjing Medical University, No. 300 Guang Zhou Road, Nanjing, 210029 Jiangsu People’s Republic of China; 4 Department of Medical Informatics, School of Biomedical Engineering and Informatics, Nanjing Medical University, Nanjing, 211166 Jiangsu People’s Republic of China

**Keywords:** Prediction model, Readmission, Diabetes, Machine learning

## Abstract

**Background and objectives:**

Diabetes mellitus is a major chronic disease that results in readmissions due to poor disease control. Here we established and compared machine learning (ML)-based readmission prediction methods to predict readmission risks of diabetic patients.

**Methods:**

The dataset analyzed in this study was acquired from the Health Facts Database, which includes over 100,000 records of diabetic patients from 1999 to 2008. The basic data distribution characteristics of this dataset were summarized and then analyzed. In this study, 30-days readmission was defined as a readmission period of less than 30 days. After data preprocessing and normalization, multiple risk factors in the dataset were examined for classifier training to predict the probability of readmission using ML models. Different ML classifiers such as random forest, Naive Bayes, and decision tree ensemble were adopted to improve the clinical efficiency of the classification. In this study, the Konstanz Information Miner platform was used to preprocess and model the data, and the performances of the different classifiers were compared.

**Results:**

A total of 100,244 records were included in the model construction after the data preprocessing and normalization. A total of 23 attributes, including race, sex, age, admission type, admission location, length of stay, and drug use, were finally identified as modeling risk factors. Comparison of the performance indexes of the three algorithms revealed that the RF model had the best performance with a higher area under receiver operating characteristic curve (AUC) than the other two algorithms, suggesting that its use is more suitable for making readmission predictions.

**Conclusion:**

The factors influencing 30-days readmission predictions in diabetic patients, including number of inpatient admissions, age, diagnosis, number of emergencies, and sex, would help healthcare providers to identify patients who are at high risk of short-term readmission and reduce the probability of 30-days readmission. The RF algorithm with the highest AUC is more suitable for making 30-days readmission predictions and  deserves further validation in clinical trials.

## Background

The vast majority of patients with diabetes mellitus (DM), a major non-communicable chronic disease, require repeated hospitalizations due to poor disease control. The term “readmission” refers to the readmission of a patient to the same department within a certain period due to the same disease after discharge. An accidental readmission is caused by many reasons, including improper initial diagnosis, relapse, premature discharge, and others [1, 2]. The 30-days readmission rate after an index hospitalization has become an important hospital performance measure used by the Centers for Medicare and Medicaid Services and is receiving increased scrutiny as a marker of poor patient care [3, 4]. In 2014, a record-breaking fine was issued to 2610 hospitals by the Centers for Medicare & Medicaid Services because too many patients had been readmitted to the hospital within a short period of time. Accidental readmission not only increases patient financial burden but also leads to a repeated waste of medical resources. In 2017, the estimated cost of diagnosed diabetes in the United States was about $327 billion, of which $237 billion was direct medical costs [5–8]. Rowley et al. [9–11] recently estimated that the prevalence would increase by 54% between 2015 and 2030, reaching a total cost of over $622 billion. Nevertheless, compared with the overall 30-days readmission rate of inpatients [12, 13], those who were diagnosed with DM have a much higher readmission rate (14.4–22.7%). In addition, based on Agency for Healthcare Research and Quality Nationwide Inpatient Sample data from 2012, if a modest 5% reduction can be achieved, there would be far fewer admissions per year at an estimated annual cost savings of $1.2 billion [14, 15]. Undoubtedly, readmission plays an essential role in the increasing hospital-related costs and is becoming more common among elderly DM patients; as a result, DM readmissions become a growing and costly economic burden on both patients and public finance budgets, thus deserving our intensive attentions.

In clinical settings, real-world data from electronic health records (EHRs) can have more potential value than recording disease purely. The possibility of accidental readmission can be predicted accurately by analyzing the EHRs of patients who require repeated readmission and identifying their characteristics, at which time the limited medical resources can be reserved for the patients who need them the most. In addition to effective predictive models, identifying features associated with risk factors related to readmission in medical records will enable more careful and effective treatments in future. To manage this concerning issue, practical models that can precisely predict the possibility of individual readmission are critical. Therefore, this study analyzed the medical records of 100,244 diabetes patients in the US Health Facts Medical Database from 1999 to 2008. The risk factors were analyzed and multiple machine learning (ML) algorithms were applied to build prediction models for diabetic patients at higher risk of readmission using their multivariate medical records.

## Materials and methods

### Dataset

The data analyzed were acquired from the Health Facts Database (Cerner Corporation, US), which includes 130 hospitalized medical records of diabetes patients from 1999 to 2008. A total of 55 related attributes were included, such as admission times, sex, age, admission type, length of hospital stay, number of laboratory tests, glycosylated hemoglobin results, diagnosis, and medication. The dataset consisted of clinical records of diabetic inpatients with a length of 1–14 days hospital stay, and laboratory tests as well as medications used during hospitalization [16].

### Characteristics of the diabetes dataset

**Fig. 1:**
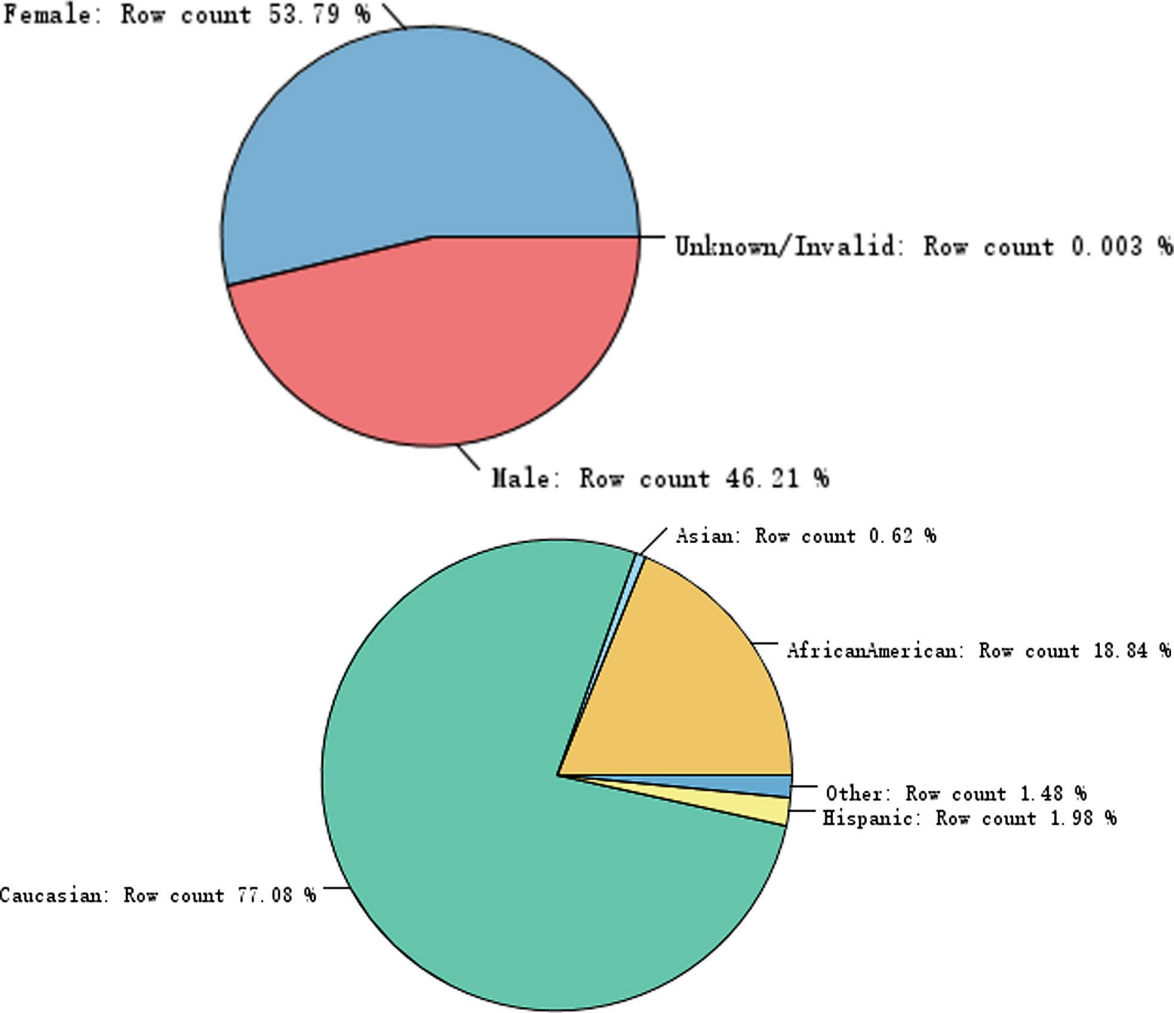
The distribution of gender (up) and race (down) in the dataset

**Fig. 2:**
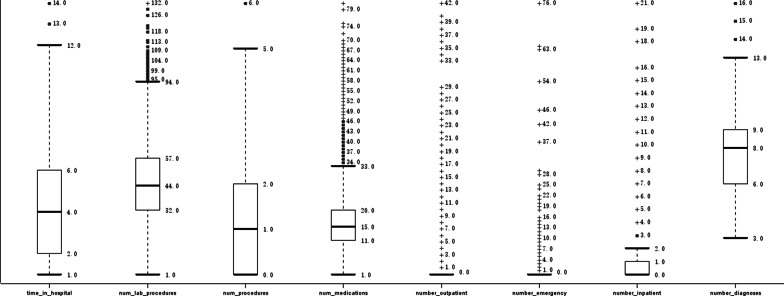
Basic characteristics of numerical parameters in records

**Fig. 3:**
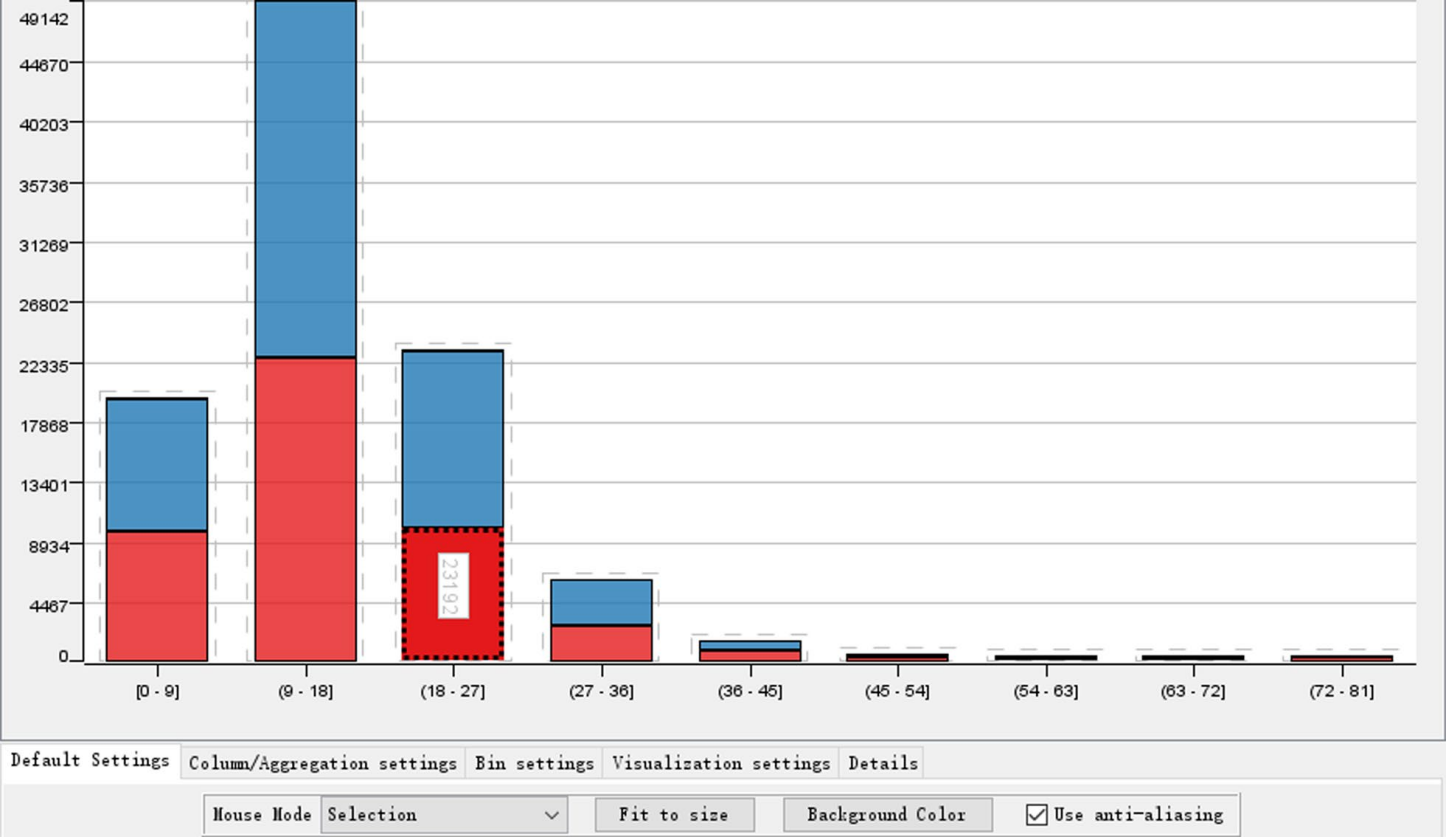
Gender distribution of medication use

The patients’ general demographic data, such as sex, age, and race as well as the clinical records of drug use, clinical operations, admission times, and others were analyzed as shown below. Nearly half (46.15%) of the total patients were male, while the majority of patients (76.49%) were white Americans (Fig. 1). The statistical analysis of the patients’ medical data revealed that most of the recorded data were distributed in an unbiased manner, despite of some outliers for some of these attributes, such as the number of emergency admissions of patients exceeding 70 times (Fig. 2), which might represent an individual extreme case or data errors. The records of male patients were marked as green, whereas those of female patients were marked as red. The numbers of doses and patients were plotted on the abscissa. Figure 3 showed that, with increasing drug use, the number of patients decreased significantly; however, there was no significant difference in gender proportion, while the largest number of patients used 9–18 different medications.

**Fig. 4:**
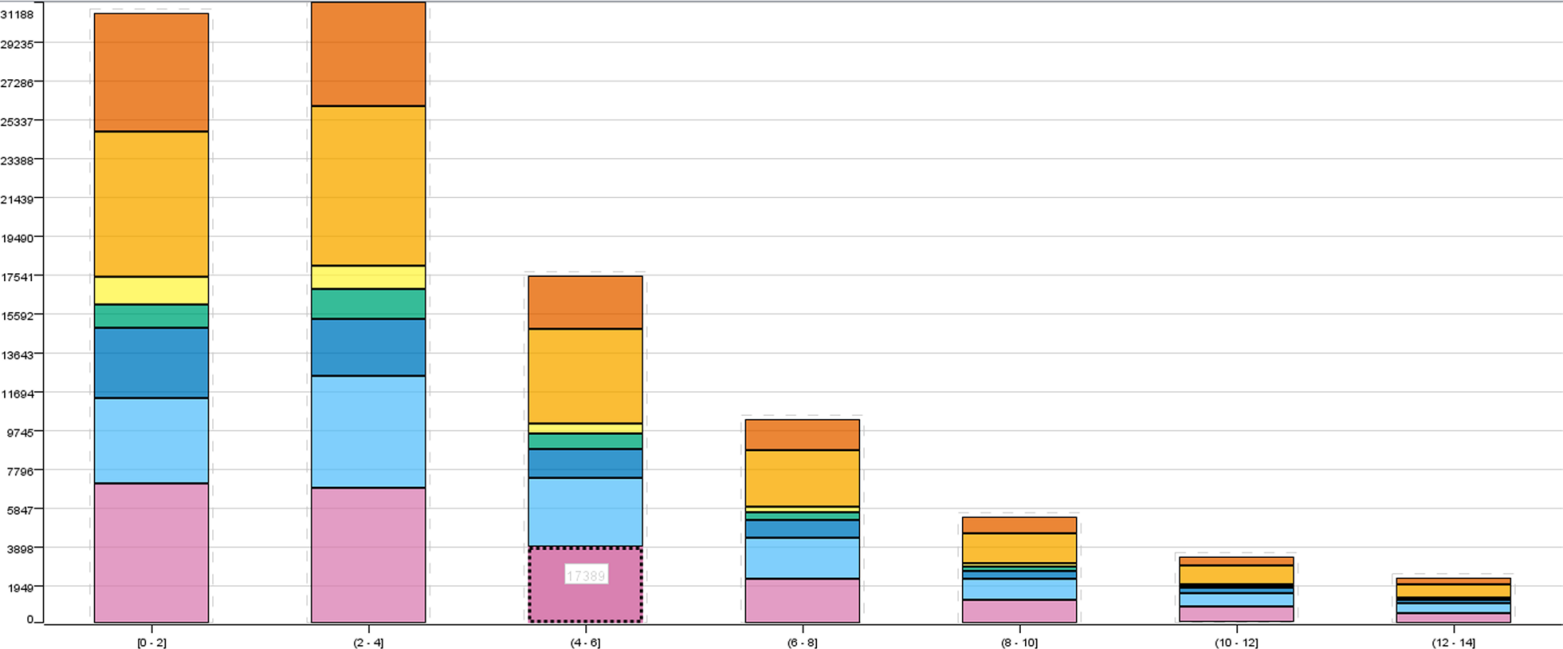
Length of hospital stay among patients at different ages

**Fig. 5:**
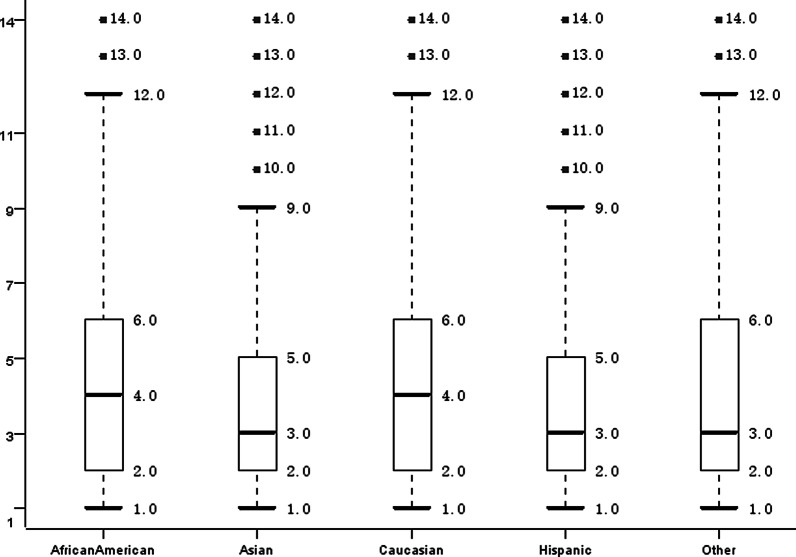
Length of hospital stay among patients with different races

The histogram of the age distribution with different length of stay was demonstrated (Fig. 4). There were a total of 8329 patients aged between 70 and 80 years with a hospitalization period of > 3 days and ≤ 6 days, and there were a relatively large proportion of teenagers aged between 10 and 20 years because young patients with relatively strong physical condition tended to have the shortest hospital stay. Figure 5 showed a statistical box chart of hospitalization time distribution of different races, demonstrating a mean 4-days hospitalization time of White and Black Americans in the United States, which was slightly longer than the hospitalization time of patients of other races in the dataset. Further data verification and cleaning was conducted in cooperation with other data of these patients in the subsequent analysis.

### Data cleansing and preprocessing

Before the analysis of readmission, the overall analysis and data preprocessing performed of the hospitalization conditions in the dataset revealed that values were missing for some of the dataset attributes (Table 1). At the data preprocessing stage, the missing values were processed and the attributes with large missing areas were deleted first, such as the weight attribute (97% missing rate). Meanwhile, the payment code and medical specialty attributes that were not relevant to the overall analysis were deleted. The mode substitution method was used to compensate for the missing part of race considering the 2% missing scope. Moreover, the records for which values were missing among the three diagnosis attributes were deleted. In addition, the “examide” and “sitagliptin” attributes were deleted due to the presence of only a single value each.

**Table 1:** Missing attribute values in the dataset

Attribute	Type	Description	Missing rate%
Race	Nominal	Ethnicity, including Caucasian, Asian, African American, Hispanic, and others	2
Weight	Numeric	Weight (pounds)	97
Payer code	Nominal	Integer identifiers corresponding to 23 different values	52
Medical specialty	Nominal	Doctor professionals, such as internal medicine, surgery, and family doctors	53
Diagnosis 1	Nominal	Initial diagnosis (coded as the first three digits of ICD-9), a total of 848 different values	0.5
Diagnosis 2	Nominal	Secondary diagnosis (coded as the first three digits of ICD-9), a total of 923 different values	0.5
Diagnosis 3	Nominal	Additional secondary diagnosis (coded as the first three digits of ICD-9) for a total of 954 different values	1

The dataset used in this study described each patient’s personal information, clinical treatment-related characteristics, and diagnosis-related characteristics. Before readmission analysis, the patient characteristics were analyzed and data pre-processing was performed. The target variable readmission category was mainly defined as three values (Table 2). To create a 30-days readmission prediction model, the binary classification model was constructed to identify high-risk patients who were readmitted < 30 days after discharge (including no readmission) and > 30-days admission records, which revealed that the proportion of the two outcomes in the dataset were very unbalanced. To minimize the impact of an unbalanced dataset, down- and over-sampling methods were adopted to balance the data [17, 18]_._

**Table 2:** Readmission distribution in the dataset

Readmission (days)	Description	Number	Percentage
< 30	Within 30 days after discharge	11,250	11.22
> 30	More than 30 days later	35,173	35.09
No	No readmission	53,821	53.69

The down-sampling method balanced the samples by randomly reducing the sample size of most classes in the classification. Nevertheless, important information might be lost in case of fewer features. In the Konstanz Information Miner (KNIME), the “Equal Size Sampling” node was used to down-sample the dataset. The entire records from the minority category and random samples of the majority category of the same size would have been sent back by this node. Comparison revealed that down-sampling of the training set lead to a better result. The traditional over-sampling method is adopted to increase minority category randomly by simple replication, but it usually results in model over-fitting. The Synthetic Minority Oversampling Technique (SMOTE) [19] was used to synthesize the minority category, namely by analyzing accidental readmission samples and adding new synthesis samples to the dataset. To prevent over-fitting, the SMOTE should be conducted on a training set, while the training and testing sets should be cut up before over-sampling. There were 142,430 records in the data-synthesis training set.

### Feature extraction and selection

At the initial stage of the clinical data analysis modeling, there are often hundreds of characteristic variables but only a few that are truly related to the target variables of the study. The exact intake of the characteristic variables included in the analysis will significantly improve the prediction accuracy. The indispensable characteristic variables were selected during the data preprocessing stage based on diabetes-related characteristics. After the feature selection, the irrelevant features were eliminated to improve the accuracy of the prediction model and shorten the running time through the data dimension reduction. The initial diagnosis given upon hospital admission is critical. The three disease diagnoses in this dataset were represented by the first three digits of the International Classification of Diseases, Ninth Revision (ICD-9) code in the diagnostic attribute values of diag1-diag3. Moreover, similar diagnoses were merged into 16 types of diseases according to the ICD-9 coding set at the data processing stage because the scattered diagnoses were unfavorable to the analysis [20]. Finally, a total of 23 risk factors contained in the trial, including race, sex, age, admission type, source of hospitalization, length of hospital stay, drug use, and others, were selected for further analysis.

### Model selection

In this study, three ML models were selected and compared. The random forest (RF) algorithm is a basic classification algorithm built by a decision tree (DT). Every DT is considered as a weak classifier, and the collection of responses produces a strong classifier. Each DT is relatively independent and the category of input data is judged by learning a series of binary problems, which is advanced in its easy-to-understand design, high accuracy, and good robustness. The Naive Bayes (NB) classifier is one of the most widely applied ML models. It is assumed that features are independent of each other when the target value is given, the probability of each category is calculated for the given data to be classified, and the data to be classified belongs to the category with the highest probability. An ensemble study has an advantage of high prediction accuracy, especially the ensemble learning algorithm using DT as a classifier. The DT set with the ensemble tree (ET) is an algorithm combining DT and ensemble learning technique. The output model describes an ensemble of DT models and is applied in the corresponding predictor node using the selected aggregation mode to aggregate the votes of each individual DT.

### Prediction model construction

The KNIME, an eclipse-based open source analysis platform with powerful data integration and analysis functions was utilized. The research workflows were created by connecting configured nodes to edit workflows and could be deployed on any other research networks or modify nodes as appropriate for their local data situations. The readmission rate of the DM dataset was predicted by putting 23 risk factors into KNIME with the adjustment for features and model parameters during the training progress. NB, RF and ET algorithms were selected to train and test the dataset as well as compare the performances of the different algorithms. The processed dataset was divided into an 80% training set and a 20% testing set before the training.

**Fig. 6:**
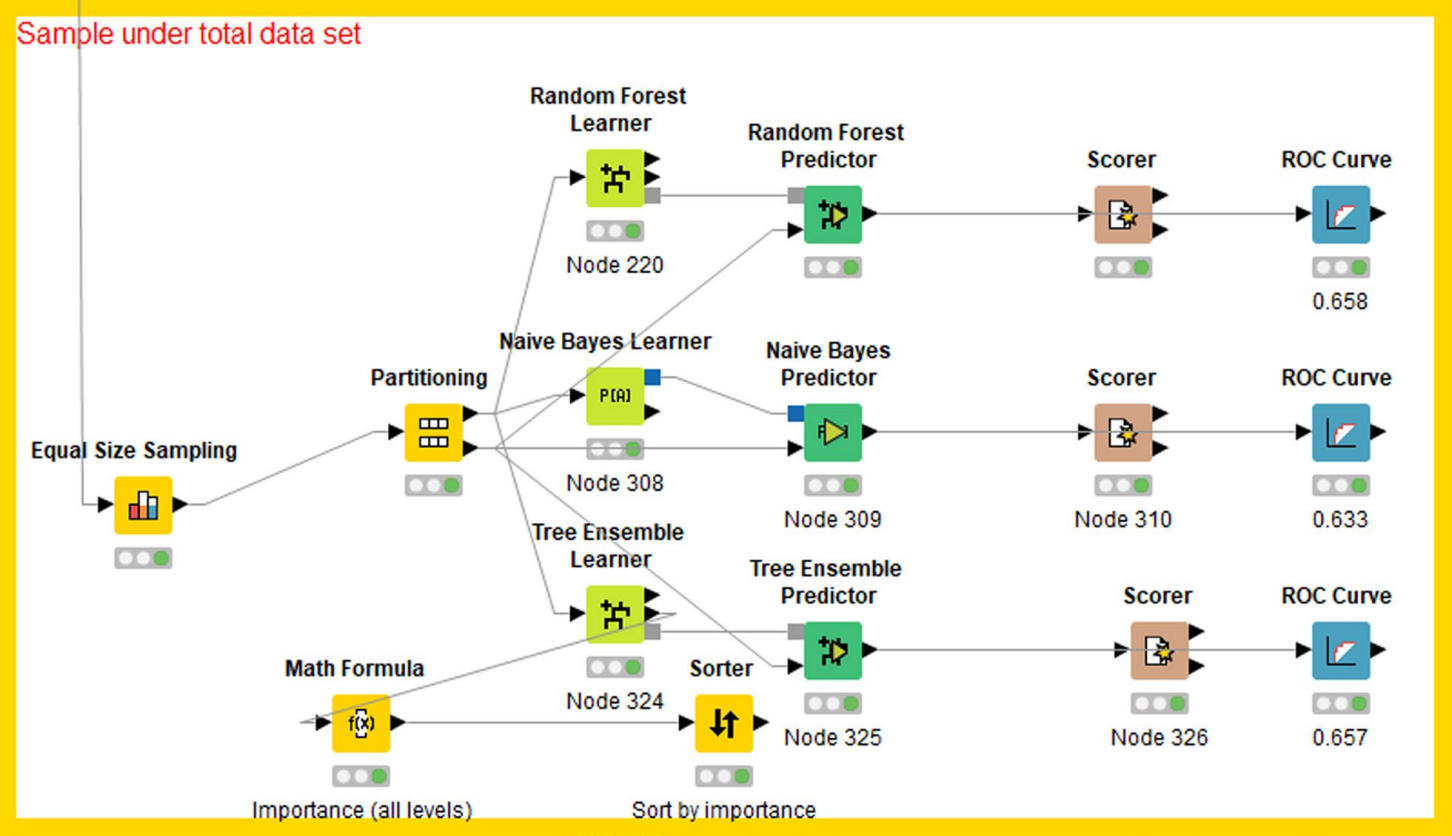
Workflow for building prediction models in KNIME

In this study, the target variable and risk factors were inputted into a Random Forest Learner node with the configured parameter in KNIME. After the training, the test set and the Random Forest Predictor node were connected to the test model (Fig. 6). The NB classification model was built on the basis of given training data by the Naive Bayes Learner node in KNIME, which calculated the number of rows per attribute value per class for nominal attributes and the Gaussian distribution for numerical attributes. After the training, the created model could be used in the NB predictor to predict the class membership of the unclassified data. The composition principle of the ET model was the same as described above (Fig. 6).

## Results

### Comparison of different model performances

**Fig. 7:**
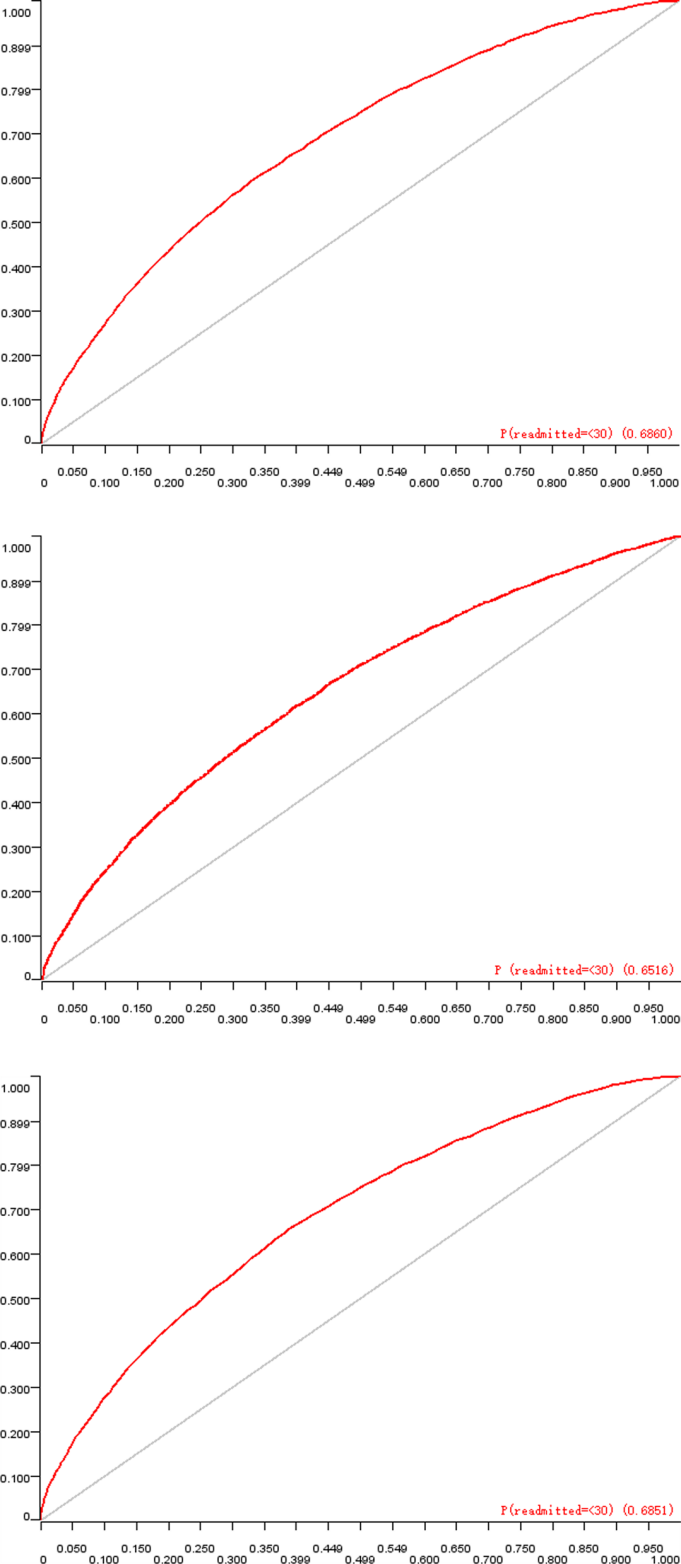
The AUC diagram of future readmission risk model based on RF, NB and TE algorithms

After data preprocessing, 100,244 records were finally included in the model construction and 23 attribute values, including race, sex, age, admission type, admission location, length of stay, and drugs used, were finally determined as included risk factors for classifier training. The area under the operating characteristic curve (AUC) value, which was used to evaluate the merits of the binary classification algorithm, was adopted as a main criterion to judge the performance of the prediction model in this study. For each type of prediction model, the mean AUC was recorded (Table 3). The down-sampling processing result was better than that of the over-sampling processing slightly in readmission within 30 days when these two methods were used to balance the dataset. In addition, the prediction of future readmission performance of patients was the best, and the RF algorithm has a higher AUC value than the other two algorithms, making it more suitable for predicting accidental readmission (Fig. 7).

**Table 3:** The performance of the different prediction models on T2D readmission

Groups	Models	Avg. AUC
30 days readmission (over-sampling)	Random Forest	0.64
	Naive Bayes	0.619
	Tree Ensemble	0.634
30 days readmission (down-sampling)	Random Forest	0.661
	Naive Bayes	0.633
	Tree Ensemble	0.659
Future readmission	Random Forest	0.686
	Naive Bayes	0.652
	Tree Ensemble	0.685

**Fig. 8:**
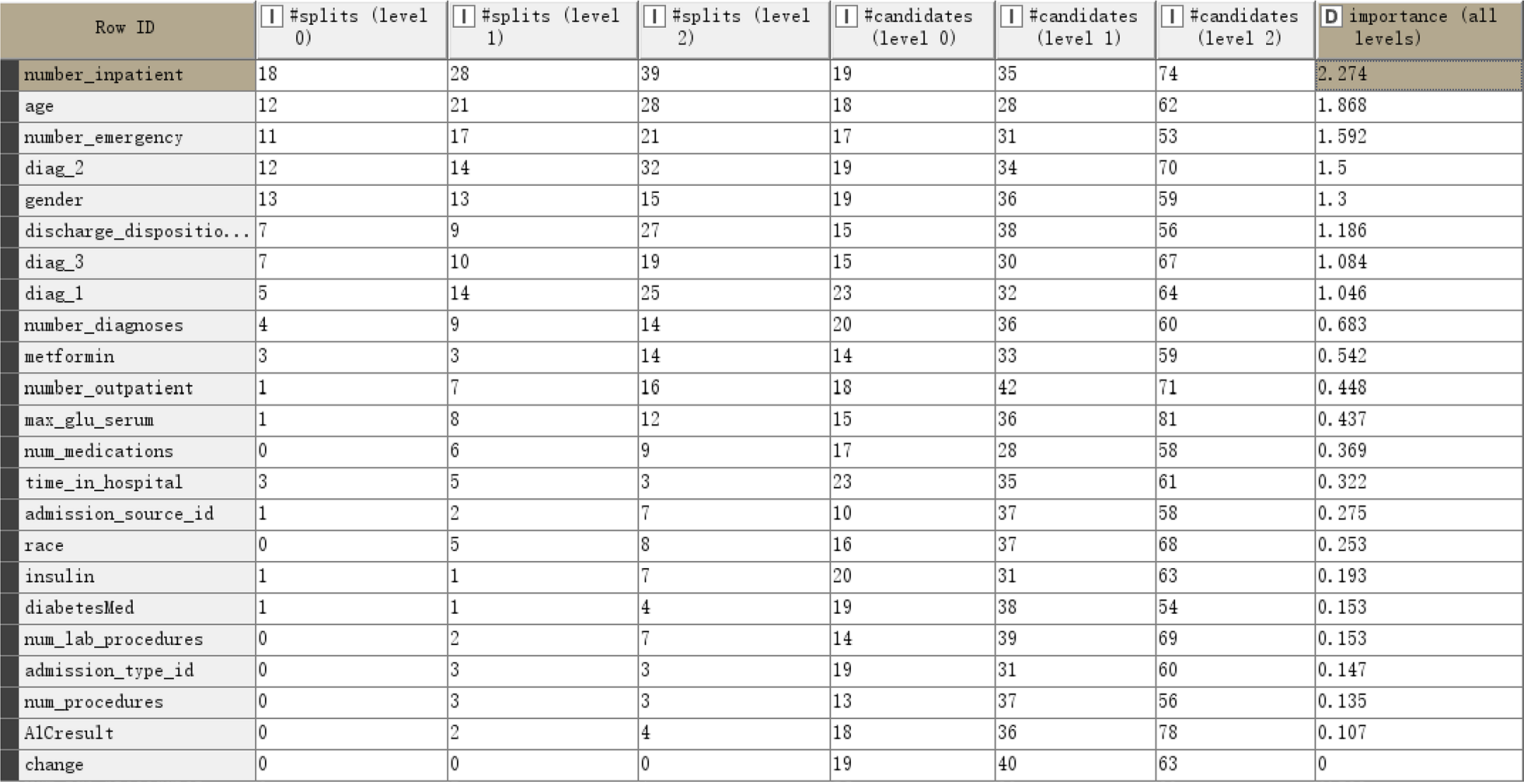
Importance of features included in future readmission prediction models

The veracity of the predictive models could be optimized by selecting most suitable features. The importance of factors in readmission prediction could be known by analyzing the variety of features in the dataset. The second output interface of the Tree Ensemble Learner node in KNIME provided particular information on the importance of features, where the frequency of building DT in the first, second, and third levels were calculated by using the features. It was adopted in this study as a method to measure the importance of features (Fig. 8).

Some patterns could be found from the order of importance of characteristics in patients with readmission. Admission times, age, diagnosis, number of emergencies, and sex were the main characteristics used to identify the probability of accidental readmission. Patients who had more admission times were the main group readmitted within 30 days and were mostly elderly. They were also more seriously ill and had longer hospital stays than the younger patients. Moreover, the elderly patients had more emergency department visits due to the sudden deterioration of their condition. Among the three diagnosis code features, the second diagnosis was more important than the first diagnosis of diag_1, indicating that the subsequent diagnosis in the EHR could reflect each patient’s condition more accurately. In summary, medical staff must provide health education and follow-up for diabetic patients with repeated admissions, especially elderly patients, to prevent the occurrence of complications and choose appropriate treatments for patients at different ages.

## Discussion

In this study, RF, NB, and TE were used to construct a 30-day hospital readmission risk model. The dataset was used to train and verify the model through an 80% training set and a 20% test set. The RF algorithm showed good predictive performance in all three models. The complete process of the model design shown here included algorithm selection, which will be of reference significance for other similar predictive model designs in the future. In a readmission risk model for patients hospitalized with cirrhosis in 2020, the AUC was 0.670 compared to existing models (0.649, 0.566, 0.577), similar to the predictive ability of the model in this study [21].

According to the model results, the number of hospitalizations, age, length of hospital stay, and sex were the main features that determined the probability of accidental readmission for predicting future readmission cases. Previous studies revealed that admission times, age, sex are relevant to multiple hospitalizations [22]. A length of stay longer than 5 days was associated with a greater than 87% risk of readmission compared to a length of stay shorter than or equal to 2 days [23]. More admission times was the main component of patients who had more readmissions, primarily elderly patients with more serious conditions, and the length of hospitalizations were much longer than that of general patients. Besides, the frequent diagnoses indicated that these patients had a higher probability of developing diabetes-related complications. Moreover, diag_2 was more important than diag_1 among the three diagnostic codes, indicating that the subsequent diagnosis in a patient’s EHR could more accurately reflect the patient’s condition. Therefore, healthcare providers must provide health education and follow-up to prevent complications for patients who was hospitalized repeatedly, especially elderly patients, which was consistent with those of Kampan [24] that inpatient education, medication adjustment, and discharge planning significantly reduced the incidence of readmission and reduce the length of hospital stay for recurrent hypoglycemia. Taking into account the scattered diagnostic codes that are not conducive to the inclusion of diagnostic factors, we improved the accuracy of the prediction model and shortened the running time by reducing the data dimension in the data pre-processing stage. Next, we will explore which diagnoses more significant impact 30-days readmission rates.

There are many classic ML algorithms in the classification of medical data. The RF algorithm can outperform the DT algorithm in most datasets, suggesting that it could be a method of feature importance computation [25]. In addition, the second output of the Tree Ensemble Learner node provides detailed information about the importance of variables that are useful for feature selection. This study also had some limitations. The distribution of the target variable was unbalanced. Most of the patients in the dataset had no readmission record (53.69%), with only 11.22% having been readmitted within 30 days (< 30), when the remaining patients (35.09%) being readmitted beyond 30 days (> 30) after the first discharge. In reality, the readmissions after > 30 days were difficult to measure because there was not much difference between admissions on day 30 and those on day 31. The reduction of overall classification accuracy is the main goal of the traditional ML algorithms. The major category gains too much attention in the process of classification when data imbalances occur, and the performance to identify minority sample decreased [26]. However, the targeting category requiring prediction is a very small proportion of the overall quantity in medical data. The inconsistency between sensitivity and specificity was significantly reduced when the training set was balanced. The DM dataset used in this study is an international public dataset with uncertain quality control, creating a major study limitation. However, it also reminds us of the importance of dataset specification in model training.

Besides, the analysis incorporates some of the factors provided in the dataset but lacks some key features, such as disease progression, family history, body mass index, and insurance information. Besides, inconsistencies existed between different genders from different races, for example, a previous study analyzed the readmission rates across non-Hispanic Whites, non-Hispanic Blacks, and Hispanics, revealing that the percentage of female patients varies among different ethnic groups [27]. In addition, the lack of practical experience of doctors at the first diagnosis and the subjective choice of patients may also account for the determined readmission rates. So many known and unknown risk factors in medical activities can affect readmissions, and model performances will be greatly improved through the analysis of real-world data and the data-driven mining of potential risk factors affecting patient readmission rates.

RF was more suitable for predicting accidental readmissions in this study. As one of the most commonly used algorithms in current classification work, RF has better predictive performance and can give variable importance measures during classification. This study adopted the algorithm encapsulated in the KNIME tool, which is relatively mature and can be convenient for clinical practitioners who are not capable of algorithm programming but want to be able to perform analyses themselves. However, there is little scope for self-modification of these algorithm parameters. Nevertheless, some measures such as the Gini index and out-of-bag data error rate for calculating the feature importance score were not considered when integrating the algorithm functions to facilitate personalized use. As an alternative, the Tree Ensemble Learner node was adopted instead of the RF algorithm to determine the importance score, which was also a limitation to this study. Moreover, our study did not exclude the planned readmissions after discharge, and researchers can adjust the training dataset as needed to predict unplanned returns. Prediction accuracy may be further improved if clinical data related to patient hospitalizations can be extended to larger sample sizes with more included features.

## Conclusion

In conclusion, ML could help healthcare providers to identify those patients who are prone to short-term readmission and might reduce the probability of readmission within 30 days by altering the risk factors.

## Supplementary Information

**Additional file 1:** The 23 risk factors included in the proposed readmission risk prediction models.

## Data Availability

The datasets adopted during the study are available in the Health Facts Database.

## Abbreviations

ML: Machine learning
RF: Random forest
NB: Naive Bayes
TE: Tree ensemble
KNIME: Konstanz information miner
AUC: Area under receiver operating characteristic curve
DM: Diabetes mellitus
EHRs: Electric health records
SMOTE: Synthetic minority oversampling technique
ROC: Receiver operating characteristic curve
